# Supplementary material for: Post Natal Microbial and Metabolite Transmission: The Path from Mother to Infant
Source: Nutrients. 2024 Jun 22;16(13):1990. doi: 10.3390/nu16131990 (PMC11243545; doi:10.3390/nu16131990)
Supplement: Supplementary file 1 [file nutrients-16-01990-s001.zip › Supplementary Figures.pdf]

Post natal microbial and metabolite transmission: the path from mother to infant

Juan Manuel Vélez-Ixta [1,†], Carmen Josefina Juárez-Castelán [1,†], Daniela Ramírez-Sánchez [1], Noemí del Socorro Lázaro-Pérez [1], José Javier Castro-Arellano [2], Silvia Romero-Maldonado [3], Enrique Rico-Arzate [2], Carlos Hoyo-Vadillo [4], Marisol Salgado-Mancilla [2], Carlos Yamel Gómez-Cruz [2], Aparna Krishnakumar [1], Alberto Piña-Escobedo [1], Tizziani Benitez-Guerrero [1], María Luisa Pizano-Zárate [5,6,*], Yair Cruz-Narváez [2,*] and Jaime García-Mena [1,*]

[1] Departamento de Genética y Biología Molecular, Cinvestav, Av. Instituto Politécnico Nacional 2508, Mexico City 07360, Mexico; juan.velez@cinvestav.mx (J.M.V.-I.); carmen.juarez@cinvestav.mx (C.J.J.-C.); danielaramireza23@gmail.com (D.R.-S.); noemi.lazaro@cinvestav.mx (N.d.S.L.-P.); aparna.krishnakumar@cinvestav.mx (A.K.); apinae@cinvestav.mx (A.P.-E.); tizziani.benitez@cinvestav.mx (T.B.-G.)

[2] Laboratorio de Posgrado e Investigación de Operaciones Unitarias, Escuela Superior de Ingeniería Química e Industrias Extractivas, Instituto Politécnico Nacional, Mexico City 07738, Mexico; jjcastro@ipn.mx (J.J.C.-A.); ericoarz@yahoo.com (E.R.-A.); marisol.salgado.mancilla98@gmail.com (M.S.-M.); cyamelgmz@outlook.com (C.Y.G.-C.)

[3] Unidad de Cuidados Intermedios al Recién Nacido, Instituto Nacional de Perinatología, Secretaría de Salud, Mexico City 11000, Mexico; silviarmzeta@yahoo.com.mx

[4] Departamento de Farmacología, Cinvestav, Av. Instituto Politécnico Nacional 2508, Mexico City 07360, Mexico; citocromo@cinvestav.mx

[5] Coordinación de Nutrición y Bioprogramación, Instituto Nacional de Perinatología, Secretaría de Salud, Mexico City 11000, Mexico

[6] Unidad de Medicina Familiar No. 4, Instituto Mexicano del Seguro Social, Mexico City 06720, Mexico

* Correspondence: pizanozarate@yahoo.com (M.L.P.-Z.); ycruzn@ipn.mx (Y.C.-N.); jgmena@cinvestav.mx (J.G.-M.)

† These authors contributed equally to this work.

Additional File 1: Figures.

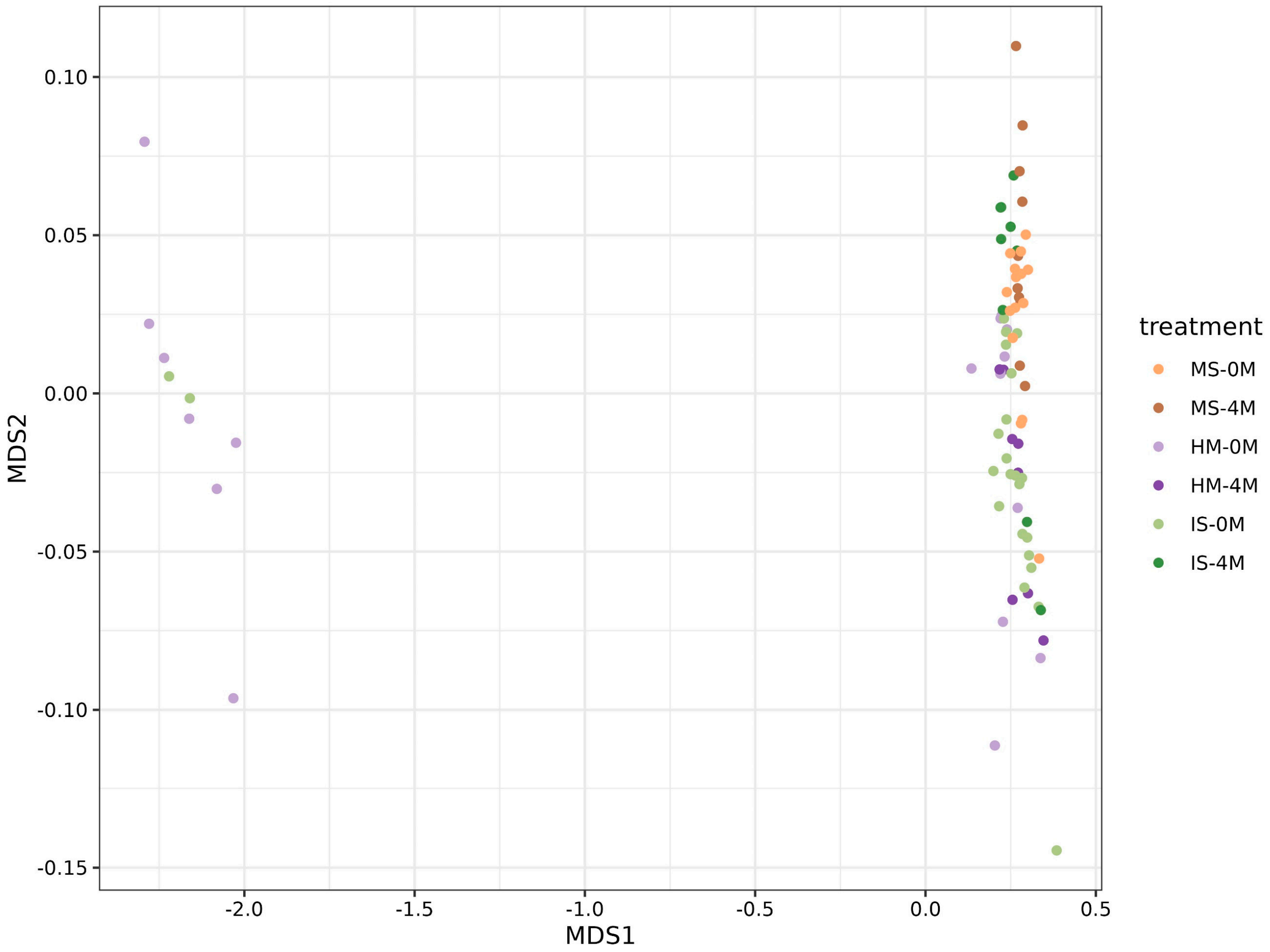

Figure S1 Beta diversity ordination scatter plot calculated with NMDS (Non-Metric Multidimensional Scaling) from weighted Unifrac distance. Each point in the graph represents a sample. MDS1 and MDS2 are represented in X and Y-axis respectively.

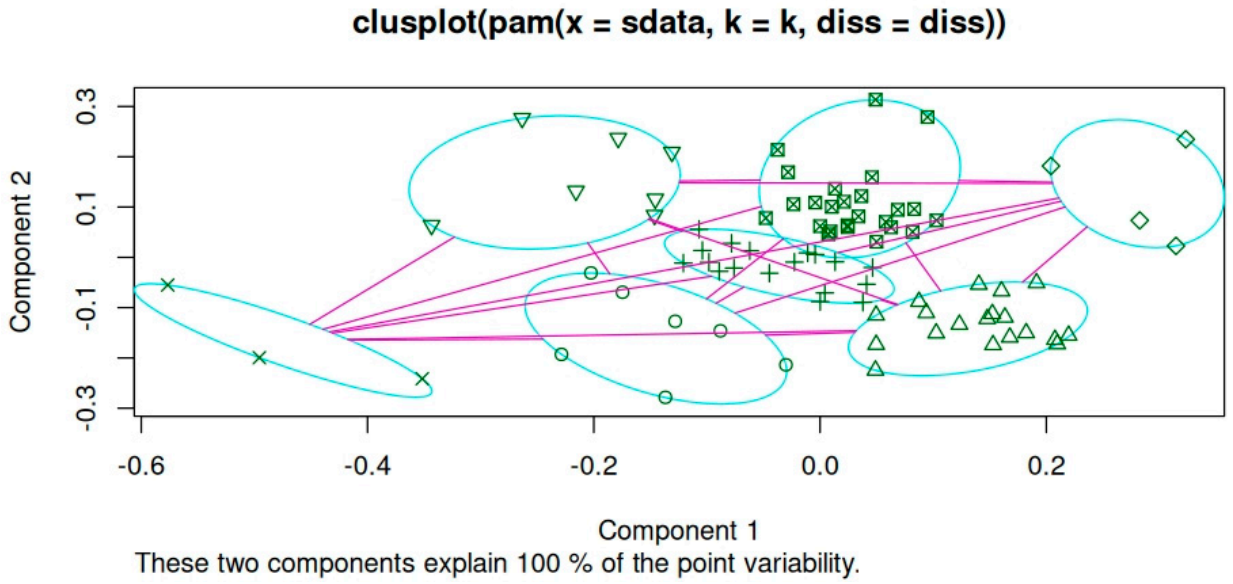

Figure S2 Clustering of samples according to their beta diversity (Unifrac distance) using k-medoids or Partitioning Around Medoid (PAM) method. An optimal number of 7 clusters was obtained with silhouette method (See Additional File 1: Fig. S3). Components explaining 100% of total variance are represented in X and Y-axis respectively.

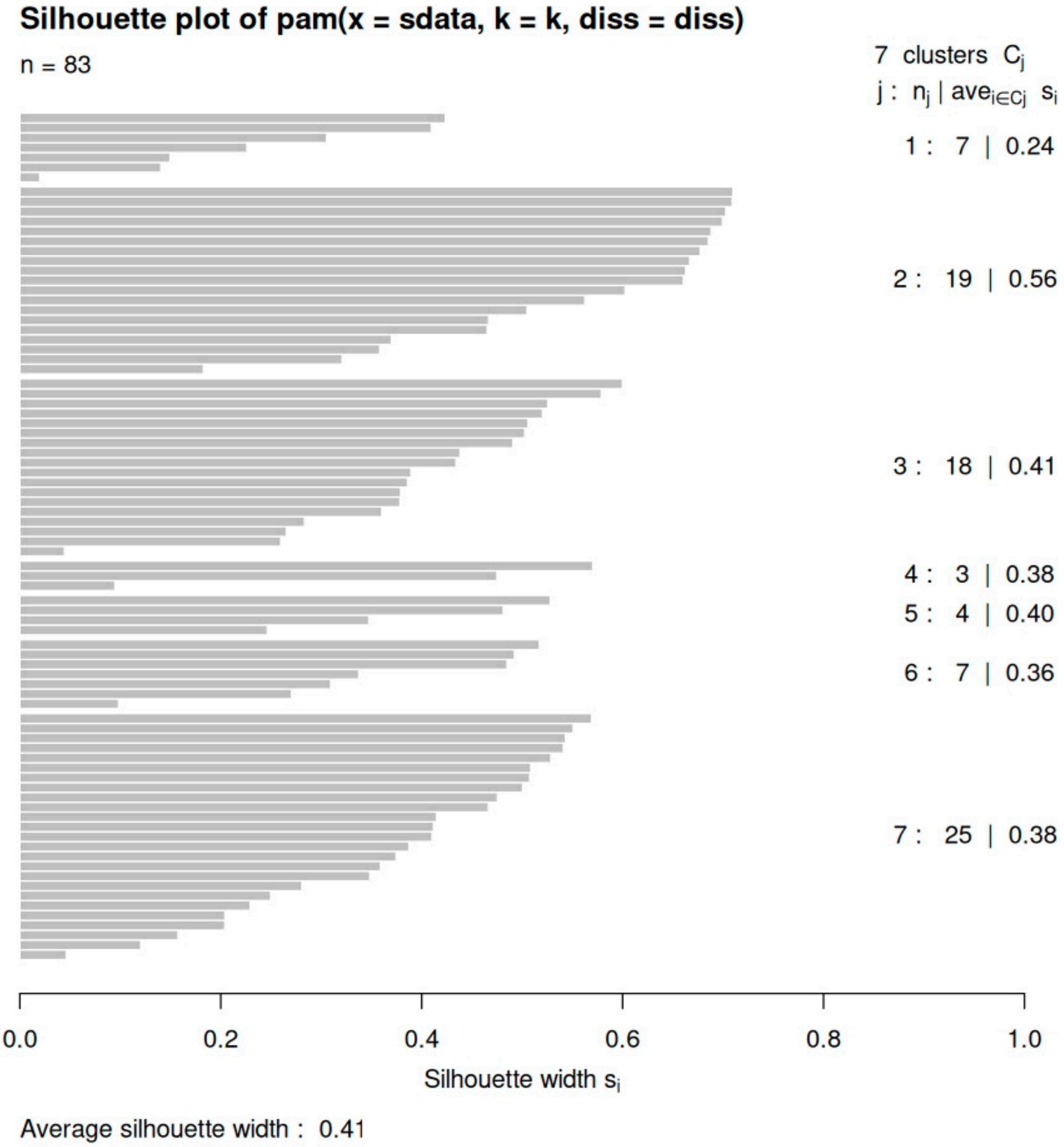

Figure S3 Average silhouette width used for optimal number of clusters calculation. A total of 7 clusters was obtained by the algorithm. The graph depicts the number of individuals composing each cluster in the Y-axis, and the respective silhouette width in the X-axis. Right side data indicate the details for each cluster.

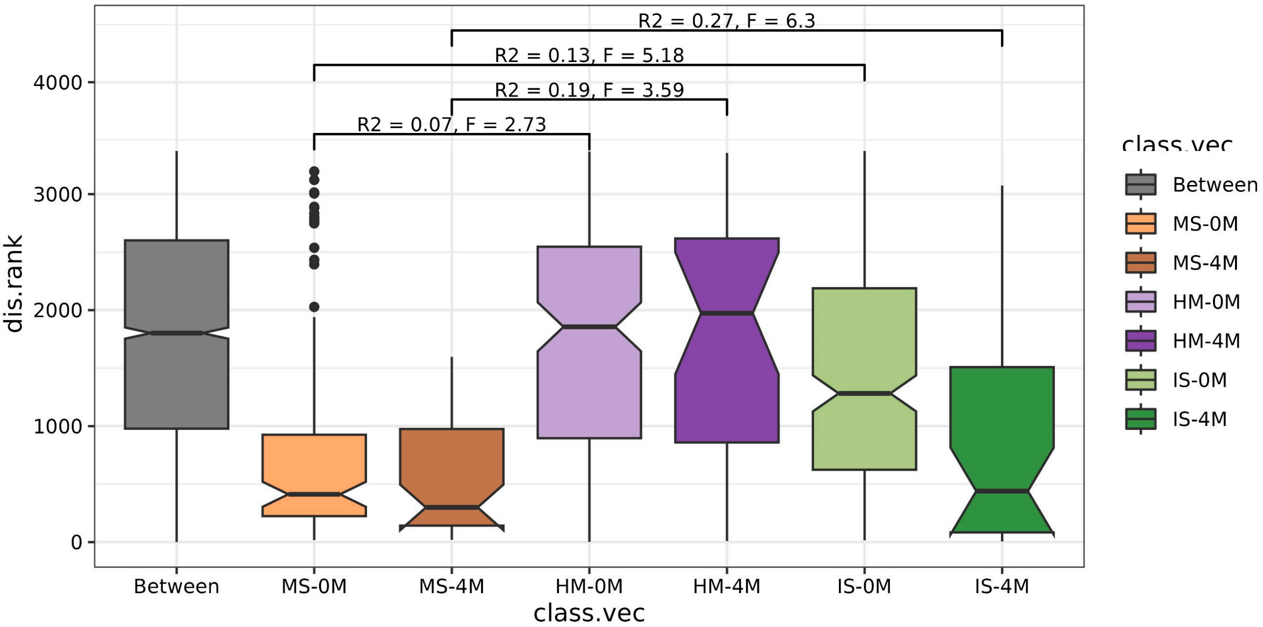

Figure S4 Notched boxplot summarizing the Analysis of Similarities (ANOSIM). Grey boxplot depicts "between-group" rank dissimilarities, the remaining represent "within-group" rank dissimilarities. ANOSIM statistic R = 0.2606, *p*-value = 0.001. Pairwise Permutational multivariate analysis of variance (PERMANOVA), also known as ADONIS *post-hoc* was performed to find where the differences occurred. Annotations in the graph show R^2 and F statistics for PERMANOVA on *q*-values < 0.05. The Y-axis shows the dissimilarity rank, the X-axis shows the class vector.

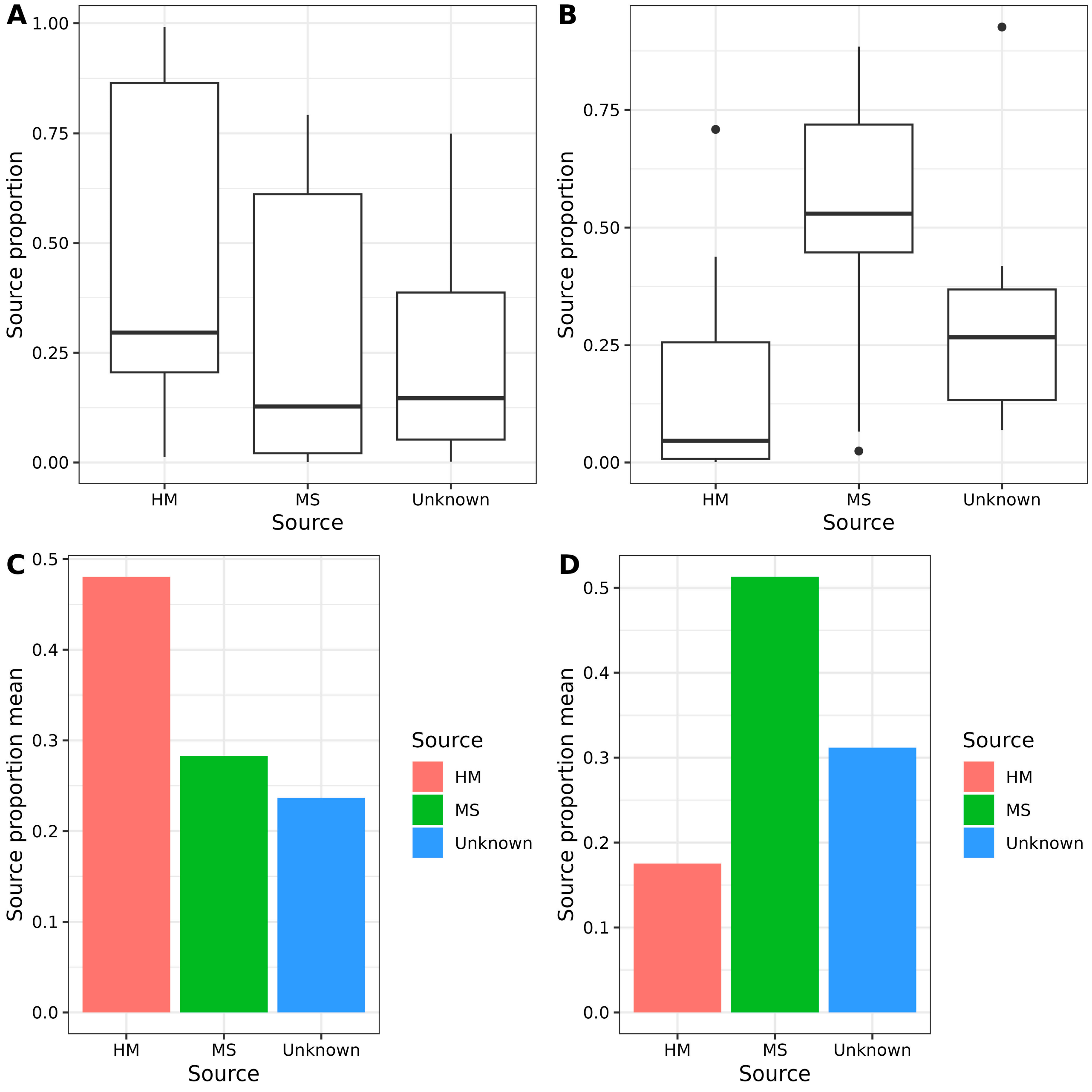

Figure S5 Sourcetracker analysis explaining the possible source of bacterial transmission. Boxplot of source proportion (Y-axis), and source (X-axis) at 0M (A) and 4M (B). Barplot of source proportion mean (Y-axis), and source (X-axis) at 0M (C) and 4M (D).

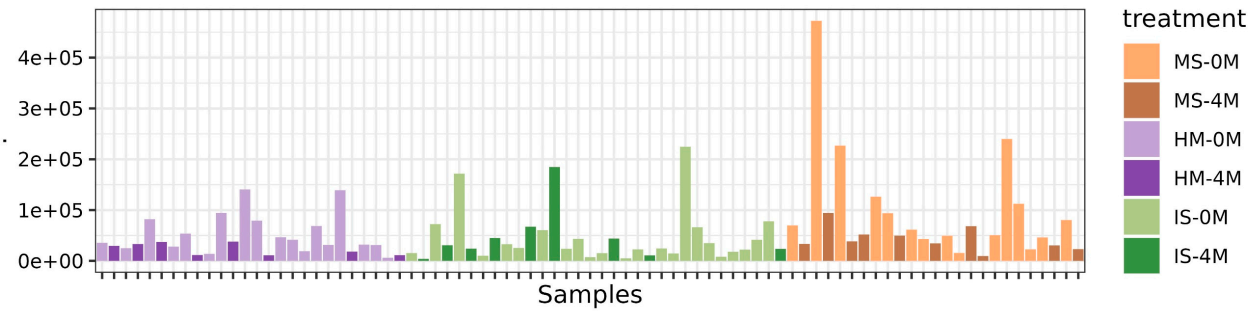

Figure S6 Sequencing depth of each sample summarized in a barplot. Samples are shown in the X-axis; counts are shown in the Y-axis. The colors indicate experimental groups, as shown in the right-side tags.

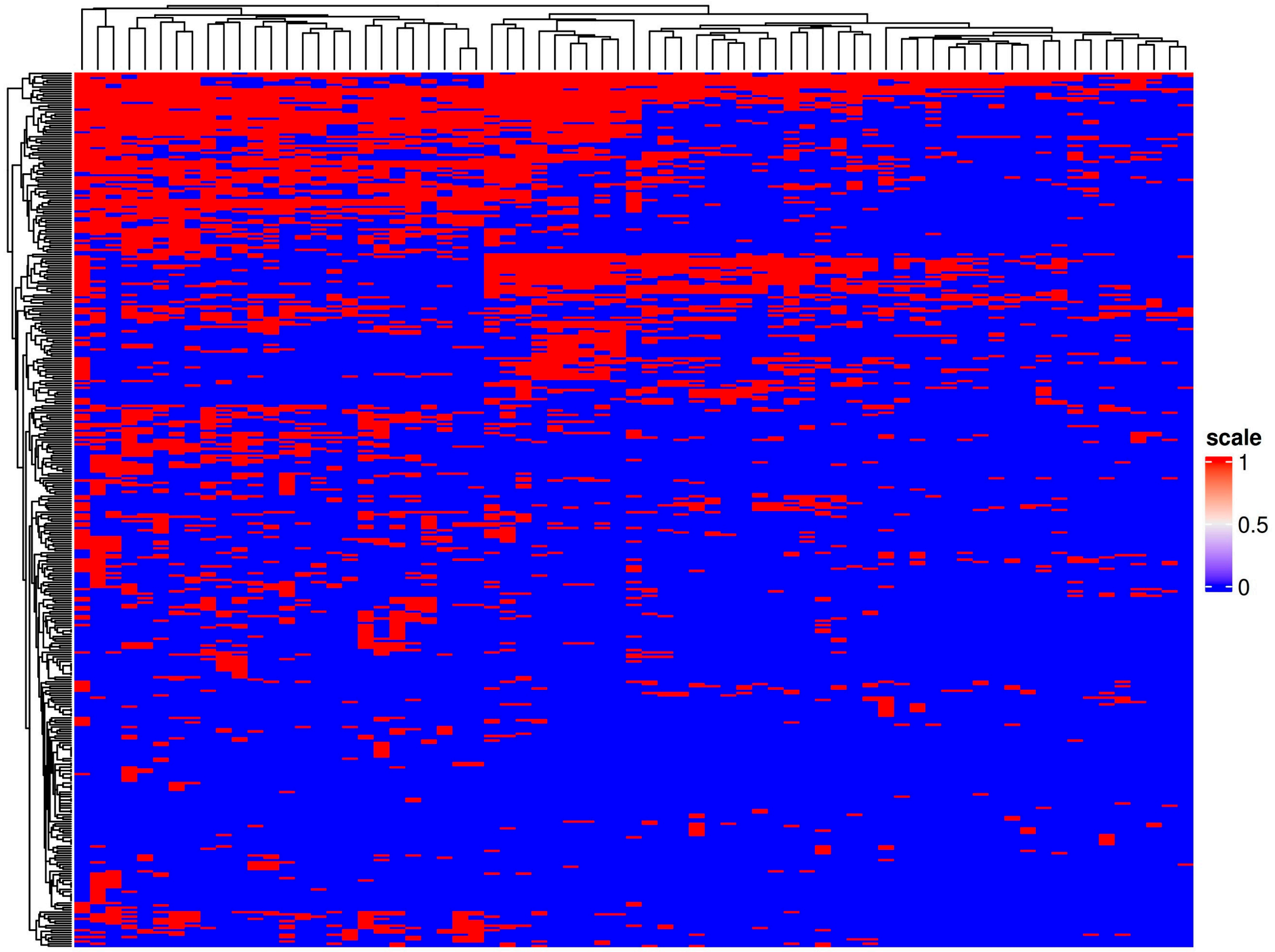

Figure S7 Amplicon Sequence Variant (ASV) presence/absence matrix plotted as a heatmap. Samples are shown in the heatmap columns, and ASVs are shown in the rows. Red color indicates the feature is present, while blue indicates the feature is absent.

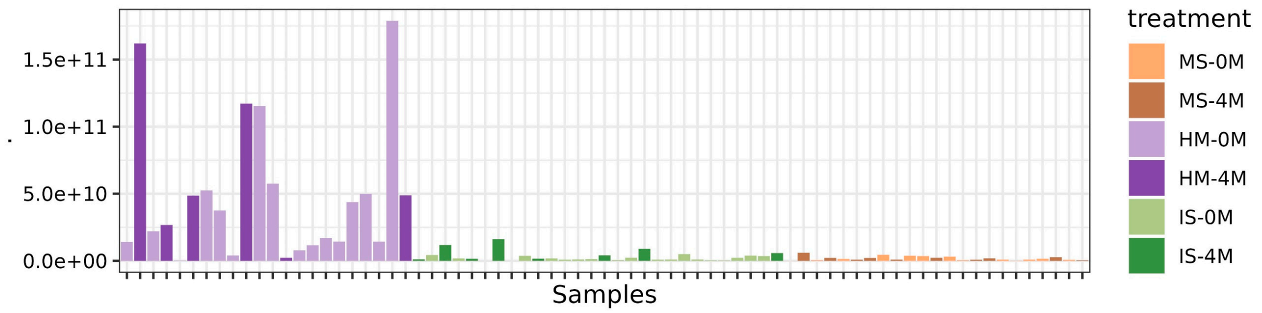

Figure S8 Total intensity of metabolites of each sample summarized in a barplot. Samples are shown in the X-axis; intensities are shown in the Y-axis. The colors indicate experimental groups, as shown in the right-side tags.

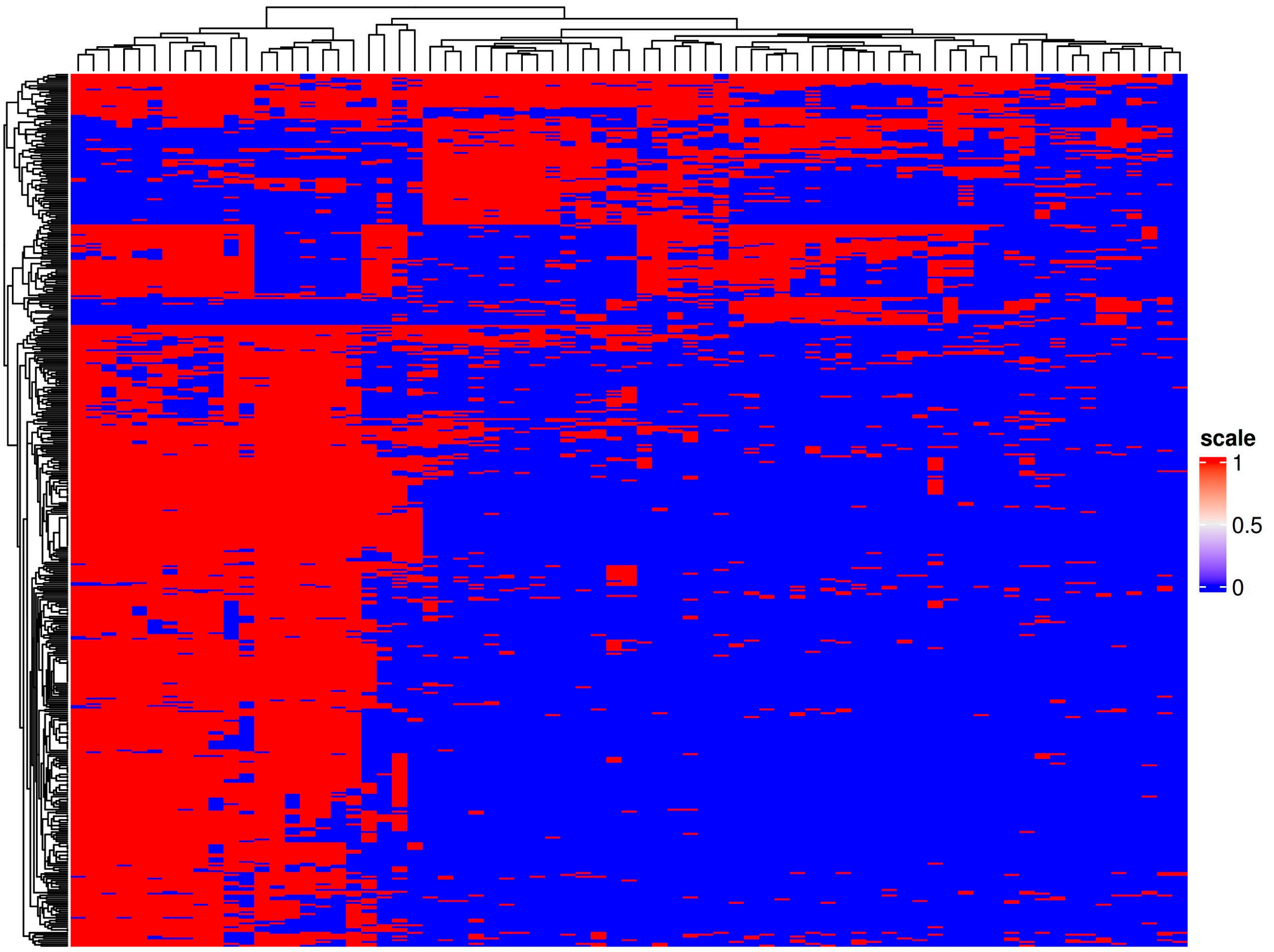

Figure S9 m/z peak presence/absence matrix plotted as a heatmap. Samples are shown in the heatmap columns, and m/z are shown in the rows. Red color indicates the feature is present, while blue indicates the feature is absent.

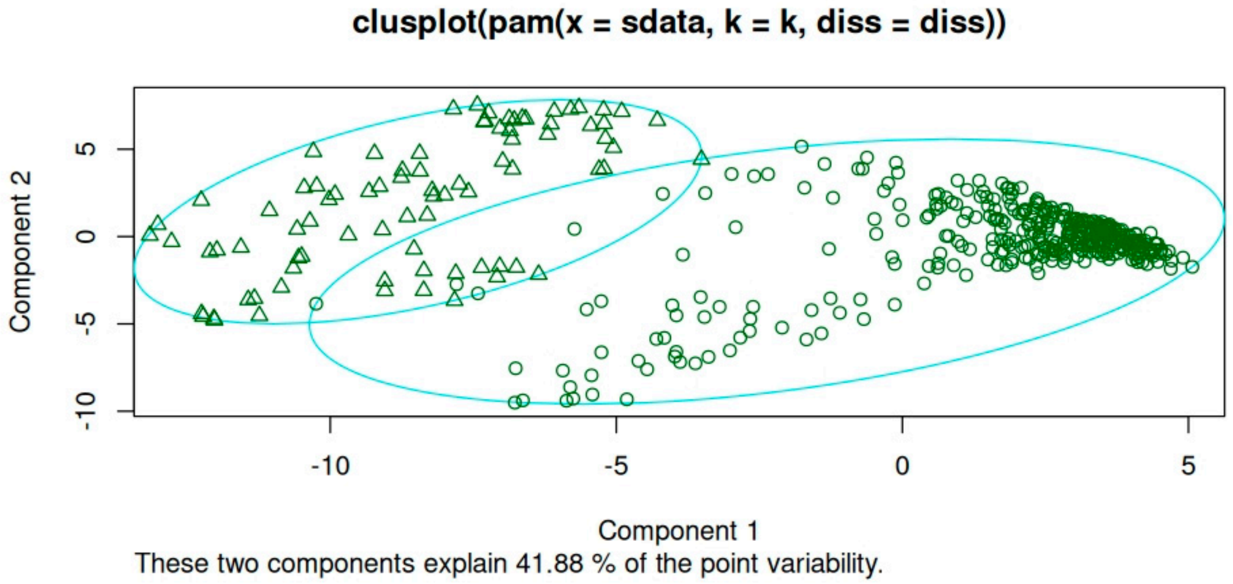

Figure S10 Clustering of m/z values using k-medoids or Partitioning Around Medoid (PAM) method, an optimal number of 2 clusters was obtained by silhouette average width method (Additional File 1: Fig. S11). Components explaining 41.88 % of total variance are represented in the X- and Y- axes.

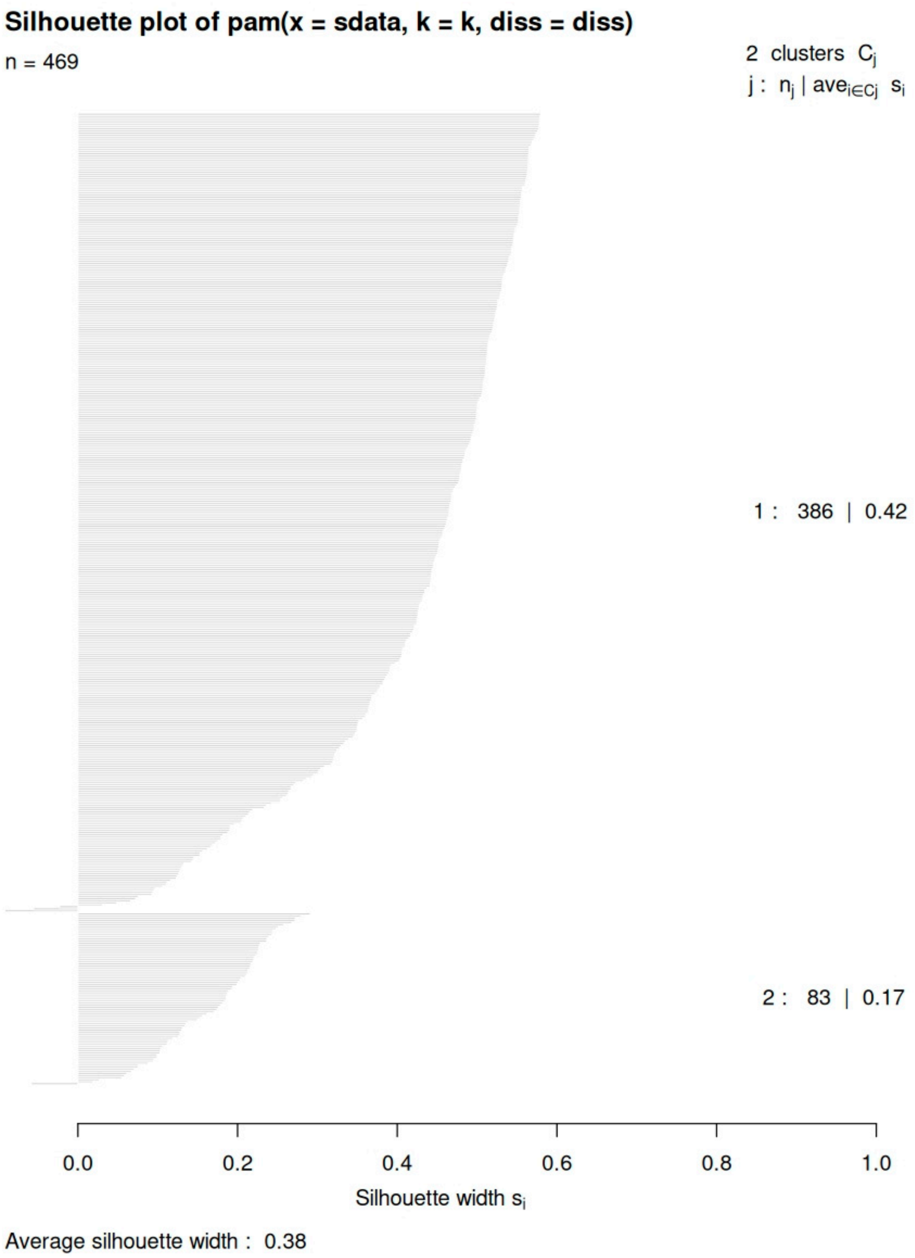

Figure S11 Average silhouette width used for optimal number of clusters calculation. A total of 2 clusters was obtained by the unsupervised algorithm. The graph shows the number of features composing each cluster in the Y-axis, and the respective silhouette width in the X-axis. Right side data indicate the details for each cluster.

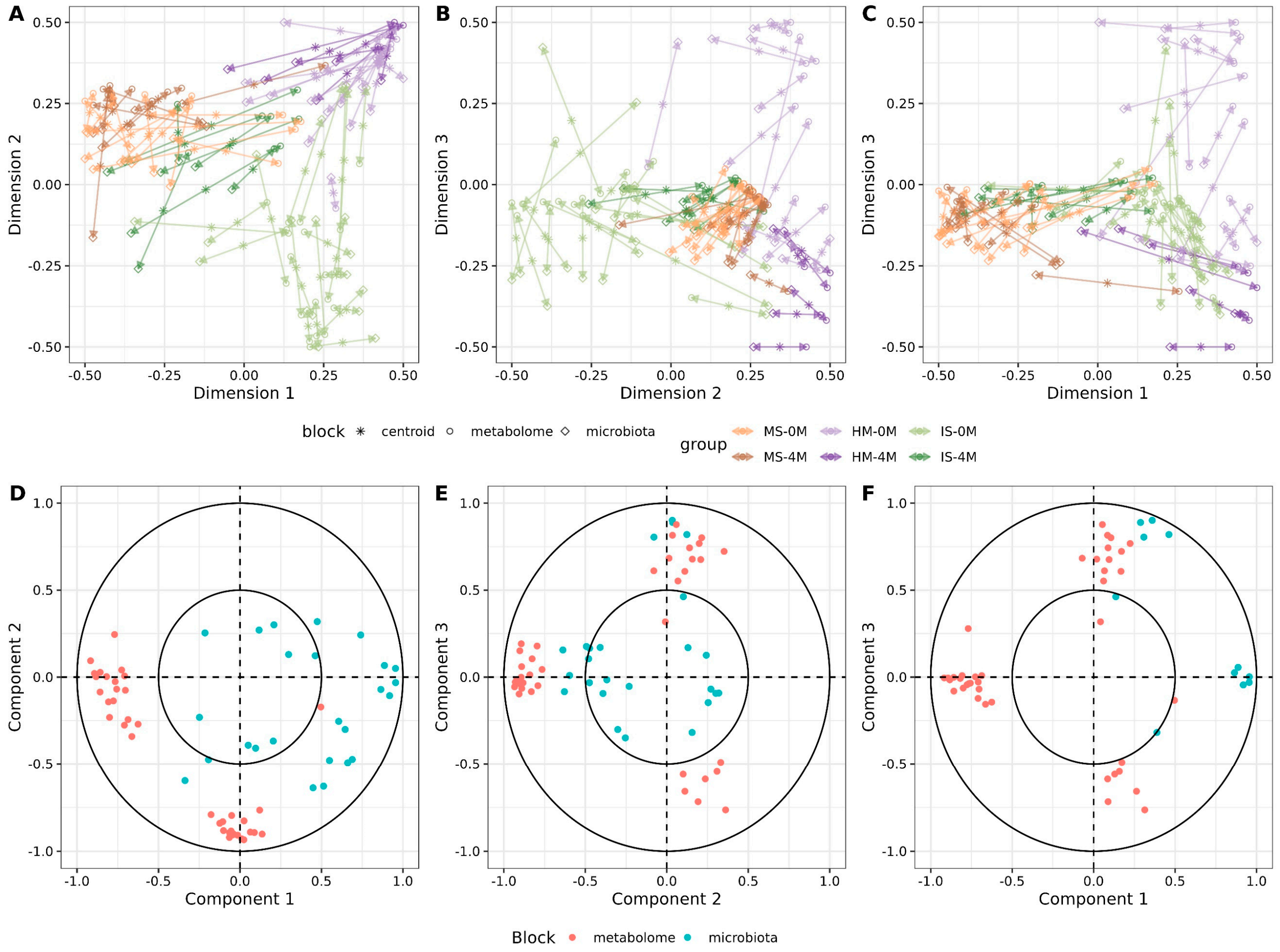

Figure S12 Representation of the Multiblock Sparse Partial Less Squares Differential Analysis (sPLSDA), also known as DIABLO of Centered Log Ration (CLR) normalized ASV and m/z matrices. For this model the set of components of Fig. 5 of the main text selected as shown in the Additional File 1: Fig. S14 and features (metabolites and bacteria) (Additional File 1: Fig. S15) was selected. (**A**) (**B**) and (**C**) are 2-D arrow plots showing the sample distribution for the microbiota and metabolome blocks in the selected pair of the three components. Symbols indicating the blocks and the color tags indicating the studied groups are shown at the bottom of the graphics. (**D**) **(E**) and (**F**) are 2-D scatter plots indicating the correlation between each sample in the selected pair of the three components.

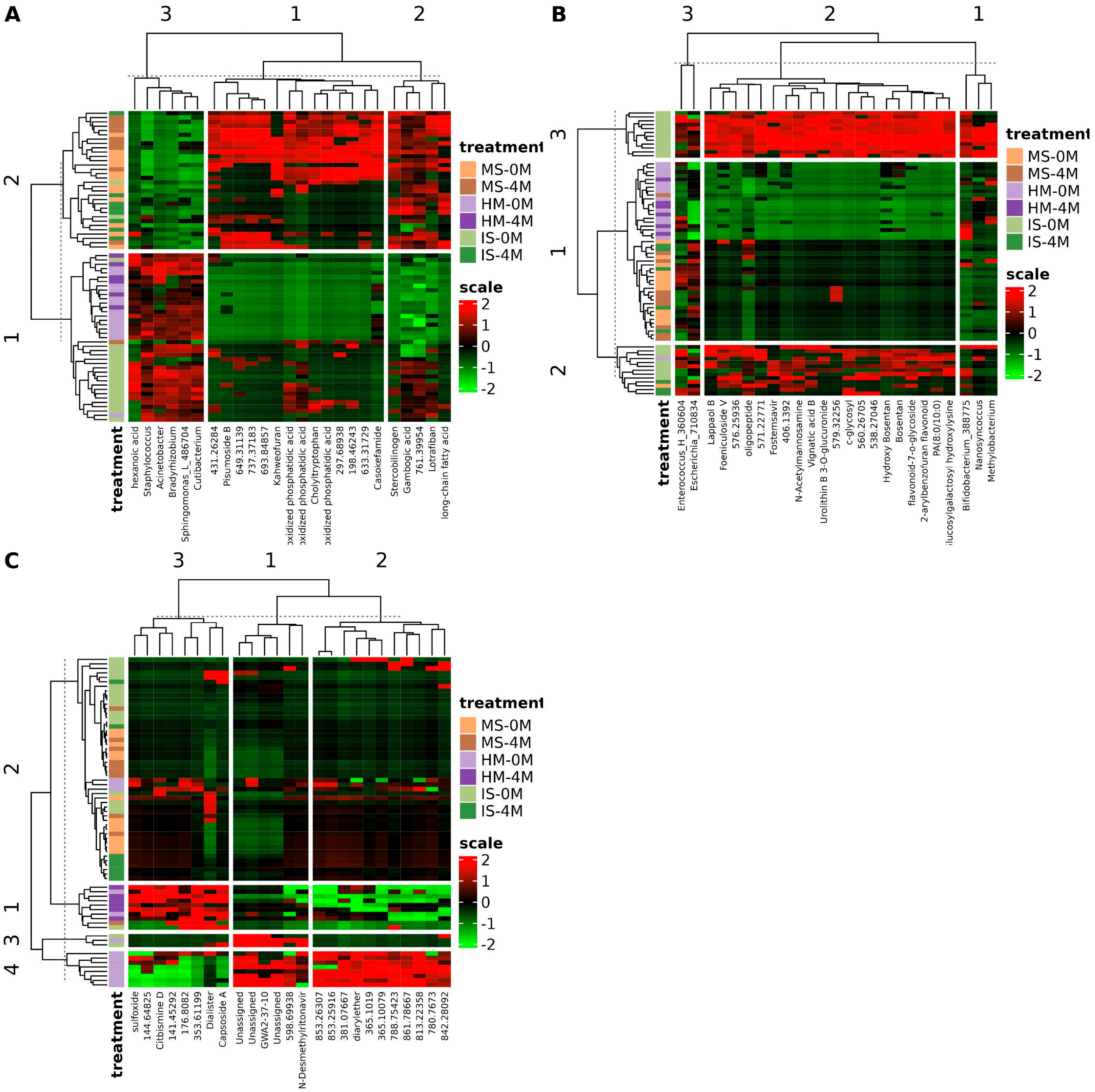

Figure S13
Hierarchical clustering heatmap summarizing main contributions in each of the (**A**) (**B**) and (**C**) components. Columns show the metabolites and bacteria, while rows depict the samples. Cuts in the dendrograms made using the k-mean method to help with the visualization are indicated by numbers on top and to the left side of the graphics. Experimental groups and heat-map scalee are shown at the right side of each heatmap.

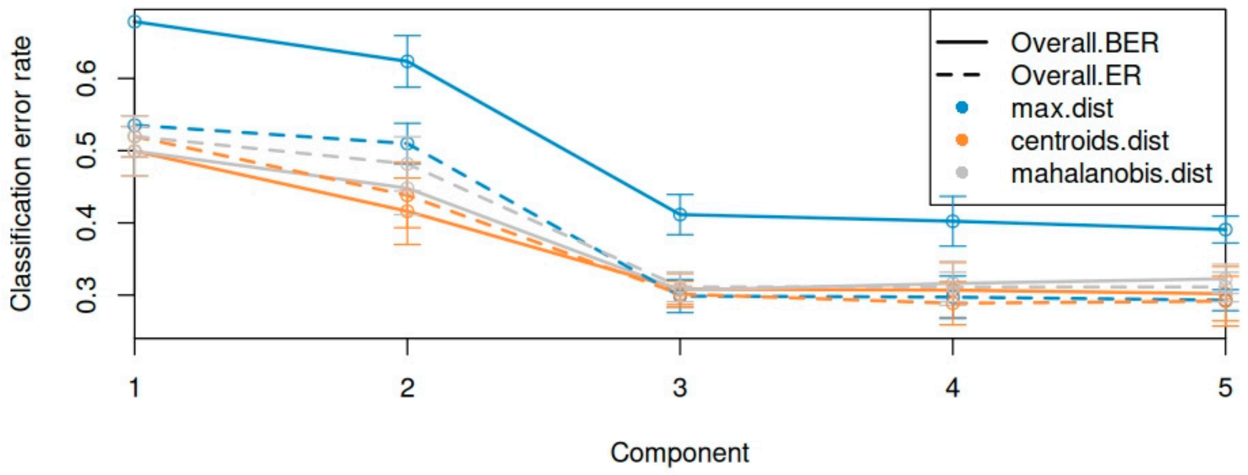

Figure S14
Graphics of the sPLSDA model performance for component (X-axis) selection, according to classification error rate (Y-axis). The first three component were selected based on this criterion.

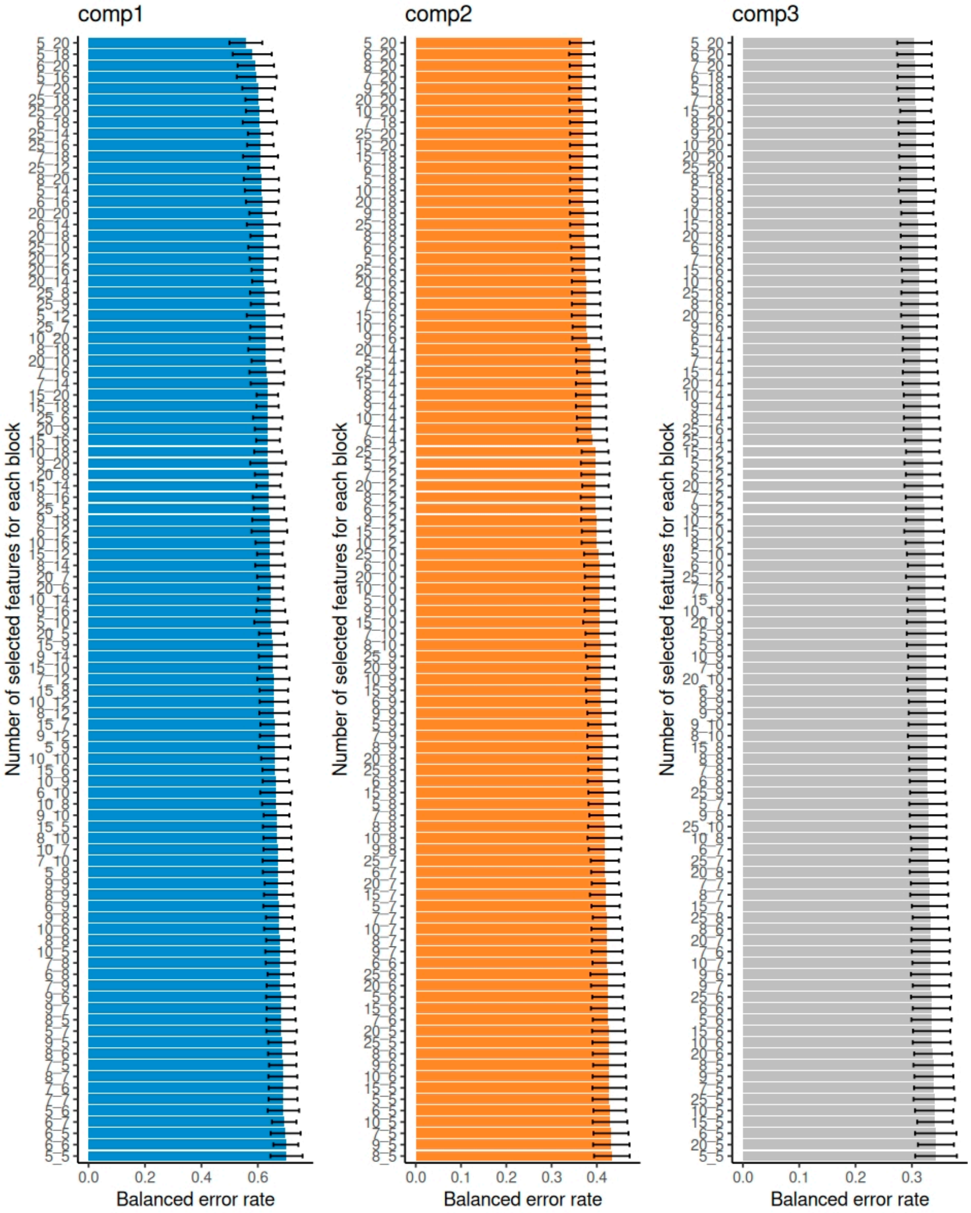

Figure S15
Graphics of the number of selected features for each 1, 2 and 3 component according to balanced error rate. X-axis represents the balanced error rate, and Y-axis shows the metabolite and bacterial features.
