# Supplementary material for: Post Natal Microbial and Metabolite Transmission: The Path from Mother to Infant
Source: Nutrients. 2024 Jun 22;16(13):1990. doi: 10.3390/nu16131990 (PMC11243545; doi:10.3390/nu16131990)
Supplement: Supplementary file 1 [file nutrients-16-01990-s001.zip › Table S2.pdf]

Table S2 Dietary data of study participants

Macronutrients and energy	Mothers 0 months (n=14)	Mothers 4 months (n=7)	p-values	W-values
Energy – kcal/day	1521.70 ± 706.13	1223.77 ± 605.24	0.3223	63.0
Total carbohydrate – g /day	220.82 ± 106.30	169.24 ± 80.19	0.3601	62.0
Total dietary fibre - g/day	20.00 ± 11.62	13.05 ± 5.10	0.2245	66.0
Total sugars - g/day	114.17 ± 48.38	84.33 ± 47.54	0.2545	65.0
Total fat - g/day	44.89 ± 25.32	38.34 ± 22.49	0.6359	56.0
Total saturated fatty acids - g/day	18.56 ± 12.59	13.89 ± 8.35	0.3223	63.0
Total monounsaturated fatty acids - g/day	14.97 ± 7.58	14.17 ± 8.06	0.9710	50.0
Total polyunsaturated fatty acids - g/day	7.30 ± 4.13	7.19 ± 4.34	0.9109	47.0
Linoleic, fatty acids polyunsaturated - g/day	5.76 ± 3.29	5.99 ± 3.49	0.8520	46.0
Cholesterol - mg/day	188.88 ± 78.53	209.12 ± 115.24	0.8557	46.0
Protein - g/day	68.41 ± 29.91	55.93 ± 26.22	0.3601	62.0
Alcohol - g/day	0.00 ± 0.00	0.01 ± 0.02	0.2217	35.5
Energy from fat - % kcal/day	26.03 ± 5.86	27.43 ± 3.39	0.4003	37.0
Energy from carbohydrates - % kcal/day	58.15 ± 8.43	55.43 ± 3.81	0.4430	60.0
Energy from protein - % kcal/day	18.34 ± 3.42	18.98 ± 2.99	0.6888	43.0
Energy from saturated fatty acids - % kcal/day	10.61 ± 3.87	9.90 ± 1.19	0.6888	43.0
Energy from monounsaturated fatty acids - % kcal/day	8.86 ± 1.66	10.15 ± 1.39	0.0861	25.5
Energy from polyunsaturated fatty acids - % kcal/day	4.17 ± 0.79	5.20 ± 1.12	0.3601	62.0
Energy from linoleic acids - % kcal/day	3.29 ± 0.56	4.36 ± 0.91	**0.0153**	16.0
Fibre, insoluble dietary fibre - g/day	8.63 ± 5.05	5.29 ± 2.11	0.1490	69.0
Crude fibre - g/day	6.55 ± 3.74	3.80 ± 1.63	0.1718	68.0
Pectin - g/day	4.44 ± 2.67	2.57 ± 1.31	0.1355	69.5

The significant p-values are in bold, The p-values and W-values are calculated using Wilcoxon Rank Sum Test.
